# Effect of total replacement of egg by soymilk and lecithin on physical properties of batter and cake

**DOI:** 10.1002/fsn3.656

**Published:** 2018-04-27

**Authors:** Sara Hedayati, Mostafa Mazaheri Tehrani

**Affiliations:** ^1^ Department of Food Science and Technology Ferdowsi University of Mashhad (FUM) Mashhad Iran

**Keywords:** cake, egg, lecithin, physical properties, soymilk

## Abstract

The baking industry is interested in finding cost‐effective and healthful alternatives for eggs. Therefore, in this study the effects of total replacement of egg by soymilk (SM) in combination with 0–6% soy lecithin (SL) on batter (density, microstructure, viscosity, and texture) and cakes (height, volume, density, texture, color parameters, and sensory attributes) were determined and compared with cakes manufactured with eggs. The results showed that all batters had shear thinning behavior and provided a good fit for the power law model. The egg‐free cake in the absence of SL was downgraded because of high density and viscosity, small air bubbles, dark color, firm texture, low volume, and sensory scores. Inclusion of up to 4% SL to the SM was found to be significant in improving cake quality and led to cakes more similar to the control sample; however, higher levels of SL had negative effects on organoleptic properties of cakes.

## 1 INTRODUCTION

Eggs are the most costly ingredients in cake formulation and have high cholesterol (Ashwini, Jyotsna, & Indrani, 2009). Thus, acquiring cost‐effective and healthy sources for partial or total replacement of egg is of great importance. Egg has several functional properties including forming a part of cake structure, coloring, flavoring, binding, foaming, tenderizing, emulsifying, and improvement of nutritive value of cake (Ashwini et al., 2009; Ratnayake, Geera, & Rybak, 2012; Wilderjans, Luyts, Brijs, & Delcour, 2013). Therefore, finding an appropriate substitute for eggs is difficult. Myhara and Kruger (1998) investigated the replacement of egg white with decolorized bovine plasma protein in cakes and found that cakes made with plasma protein had comparable volumes to those made with egg white but had darker crusts and crumb color and had an unpleasant flavor. Abdul Hussain and Al‐Oulabi (2009) in their study substituted egg with whey protein(WP) and lupine protein(LP) and found that total replacement of egg with a combination of 9.5 g LP, 16 g WP, and 5 g soy lecithin in 80 g of water resulted in reasonable cake volume. Ashwini et al. (2009) studied the effect of different hydrocolloids and emulsifiers on eggless cake quality and found that best results were obtained by hydroxypropyl methylcellulose (HPMC) in combination with sodium stearoyl lactylate (SSL). Ratnayake et al. (2012) partially substituted eggs with three commercial egg replacers and reported that they affected moisture retention, texture, color, bulk density, and flavor; however, some of these changes were not distinguished in sensory evaluation.

Soy has great potential for use in human diet due to its high level of good‐quality proteins, essential amino acids, omega‐3‐fatty acids, isoflavones, and dietary fibers (Riaz, 2005; Smith & Circle, 1972). Soy‐based ingredients are protective against breast and colon cancer, decrease triglycerides, total cholesterol, and LDL cholesterol, reduce menopausal symptoms, and prevent osteoporosis and cardiovascular diseases (Anderson, Johnstone, & Cook‐Newell, 1995; Cassidy, Bingham, & Setchell, 1994; Thiagarajan, Bennink, Bourquin, & Kavas, 1998). They also have unique functional properties. Soybean products improve textural parameters, water retention, gelling properties and extend the shelf life of food products (Majzoobi, Ghiasi, Habibi, Hedayati, & Farahnaky, 2014). Due to physiological and functional reasons, there is increasing interest in incorporation of soy in foods as partial or total replacement (Godfrey & Limpert, 2002) and one of the most promising uses of soy‐based materials is the fortification of baked goods. Lecithin is a great emulsifier that is naturally found in some foods including egg yolks and soybeans. Soy lecithin is a by‐product of soybean processing, which is widely used in food industry due to its availability, low cost, excellent emulsifying, and binding properties. It also has been shown to provide health benefits and improve protein content of foods (Szuhaj, 1989; Van Ee, 2009). The average lecithin content of fresh hen egg is 2.94% (Min, Qing‐lian, Yu‐shi, & Kuan‐wei, 2012) while dry soybeans have 1.48 to 3.08% lecithin. Hence, the amount of lecithin in soymilk with the same dry matter is much lower than eggs. Therefore, lecithin should be included to the formulation of eggless cake that contains soymilk to compensate the lower lecithin content. The main objective of the present research was to study the possibility of total replacement of egg using soymilk and soy lecithin and examine its effects on cake quality and determine the most appropriate level of lecithin.

## 2 MATERIALS AND METHODS

### 2.1 Materials

Wheat flour, fresh eggs, white fine sugar, vegetable oil, low‐fat milk, baking powder, and vanilla were purchased from local market (Mashhad, Iran). Full‐fat soy flour contained about 40% protein, 22% fat, 4% fiber, and 5.50% ash was obtained from Toos Soya Inc., Mashhad, Iran, and soy lecithin was supplied by Behpak Industrial Co., Behshahr, Iran.

### 2.2 Soymilk preparation

Soymilk was prepared by stirring the appropriate amount of full‐fat soy flour and distilled water so that suspensions contained 25% of dry matter (w/w) (equal to the dry matter of eggs) and then was blended with a kitchen mixer (Moulinex, DFC3, France) for about 10 min (Nikzade, Mazaheri‐Tehrani, & Saadatmand‐Tarzjan, 2012). Subsequently, 0 to 6% SL was added to the samples and after mixing and obtaining a homogeneous composition, the resultant soymilk was refrigerated.

### 2.3 Batter Preparation

The control cake formula included 100 g flour, 70 g sugar, 62.5 g egg, 62.5 g low‐fat milk, 30 g vegetable oil, 3 g baking powder, and 0.45 g of vanilla, and for egg‐free cakes, egg was replaced by the same weight of SM in combination with varying levels of SL.

Egg or egg substitute, vanilla, and sugar were mixed for 5 min in a Kitchen mixer (Moulinex, DFC3, France), and then, milk and vegetable oil were added to the mixture. After 2 min of mixing, flour and baking powder were added and batted slightly until a homogeneous batter was obtained.

### 2.4 Batter density

Batter density was determined at ambient temperature (22°C) by dividing the weight of a standard container filled with batter by the weight of an equal volume of water (Majzoobi, Darabzadeh, & Farahnaky, 2012).

### 2.5 Batter viscosity

Rheological properties of cake batters were measured using rotational viscometer (Bohlin Model Visco 88, Bohlin Instruments, UK) by means of C14 or C25 measuring spindles (based on viscosity of batters) at 25°C. The temperature of samples was maintained constant using a heating circulator (Julabo, Model F12‐MC, Julabo Labortechnik, Germany). Cake batter was loaded into the cup and allowed to stand for 1 min to relax and reach the desired temperature and then was subjected to a programmed shear rate, increasing linearly from 0 to 200 per s.

### 2.6 Textural properties of batter

The textural properties of batters were measured by a texture analyzer (QTS‐25, Brookfield CNS Farnell, Borehamwood, UK). Batter samples (60 ml) were poured into the extrusion cell with internal diameter of 50 mm. The back extrusion test was performed at constant speed of 50 mm/min using a cylindrical probe (42 mm diameter). The textural parameters such as firmness, consistency, cohesiveness, and index of the viscosity were calculated from the resulting force–time curves using Texture Pro Software.

### 2.7 Microstructure of batter

To study the microstructure of batter, a drop of it was transferred onto a glass slide and was covered by a coverslip taking care to prevent the inclusion of exogenous air bubbles. The slide was compressed to create a uniform layer of batter (Gómez, Doyagüe, & Hera, 2012). The prepared samples were examined using an optical microscope (Olympus BX41, Olympus Optical Co., Ltd., Tokyo, Japan) and were photographed with a microscope camera system (Olympus BP12, Olympus Optical Co., Ltd).

### 2.8 Cake baking

One hundred grams of batter was placed into a baking mold (diameter of 8.5 cm) and baked in an electronic oven at 170°C for 35 min. The baked cakes were stored in polyethylene bags at room temperature (22°C) for further analysis.

### 2.9 Cake height, volume, and density

A digital caliper was used to study the differences in height of different cakes 1 h after they were removed from the oven. Cake volume was determined using rapeseed displacement method according to the Approved Methods of the AACC (2000). The cake density was evaluated by the ratio between the cake volume and its weight.

### 2.10 Color evaluation

Cake samples were placed on a sample holder, which was at the bottom of an image‐capturing box. The digital camera was fixed 25 cm above the cakes, and fluorescent lamps inside the box were turned on 10 min before image capturing. High‐resolution pictures were taken from crust and crumb of the cakes. The images were analyzed using ImageJ software version 1.40 g (National Institutes Health, Bethesda, MD, USA) according to the following procedures:

Background contrast of the images was improved and saved in BMP format. Color space converter was used for conversion of RGB to Lab color space. Images were separated to L, a, and b‐values, where L indicates lightness, a gives redness‐greenness, and b shows blueness‐yellowness, and mean values for color parameters of crust and crumb were measured (Mohebbi, Ansarifar, Hasanpour, & Amiryousefi, 2012).

### 2.11 Determination of the textural properties of the cakes

Texture profile analysis (TPA) was performed with a Texture Analyzer (QTS‐25, Brookfield CNS Farnell, Borehamwood, UK) on cubic pieces of cake crumbs (25 mm). Tests were performed at strain deformation of 25% with constant speed of 50 mm/min and time interval of 10 s using a flat cylindrical probe (40 mm diameter). Hardness (maximum force required to compress the sample in the first bite), cohesiveness (ratio of the force areas in the second bite to the first bite), springiness (ratio of the second peak time to the first peak time), and chewiness (the result of multiplying hardness by cohesiveness by springiness) were calculated from force–time curves.

### 2.12 Sensory evaluation

Sensory hedonic test was conducted on cake samples after one‐day storage at room temperature by 12 panelists (six males and six females, age between 20 and 40 years). Panelists received training about the sensory attributes prior to sensory evaluation. Sensory characteristics including taste, crust and crumb color, texture, and overall quality were evaluated on a 1‐ to 5‐point rating scale, with score 1 to “dislike very much” and score 5 to “like very much” for each sensory parameter. The samples were served in white plastic containers in random order, and water was available for rinsing between samples.

### 2.13 Statistical analysis

All the experiments were performed at least three times. Statistical analysis was performed using SPSS software (SPSS, Inc., NJ, USA). Analysis of variance (ANOVA) was performed, and Duncan's test was employed to detect significance of differences within treatments (*p *< .05). The experimental data from viscometer were fitted to power law model by MATLAB 7 software (The MathWorks, Inc., USA) using curve‐fitting toolbox.

## 3 RESULTS AND DISCUSSION

### 3.1 Batter density and microstructure

**Figure 1:**
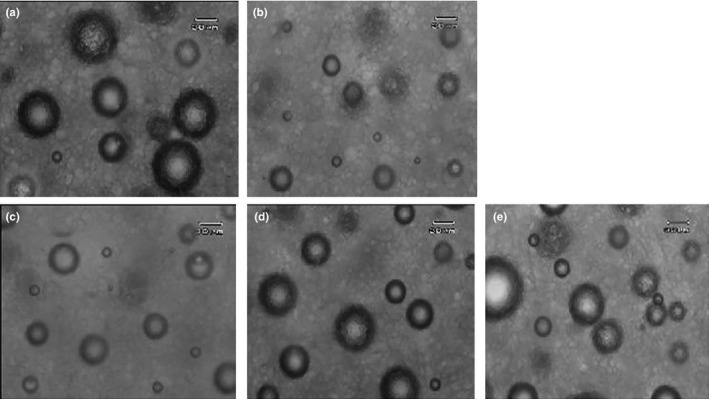
Images of cake batters structure: Control (a), SM + 0% SL (b), SM + 2% SL (c), SM + 4% SL (d), SM + 6% SL (e) . Bars on the micrographs=30 μm.

Batter density is a very important physical property as it is an indication of the amount of air bubbles incorporated to the batter depending on surface tension and viscosity of cake batter and also the mixer design and speed. Lower batter density is desired as it shows more air bubbles are incorporated into the cake batter. (Ashwini et al., 2009; Ronda, Oliete, Gómez, Caballero, & Pando, 2011). The results of batter density are presented in Table 1. Control batter had a density of 1.028 g/cc, whereas batter containing SM had a density of 1.104 g/cc. During batter mixing, egg yolk proteins decrease the surface and interfacial tensions and result in the formation of a stable emulsion (Kiosseoglou, 2004); thus, the control sample retained more air bubbles and had lower density. The egg‐free batter density decreased with addition of SL. These results coincide with observations of light microscopy, which are presented in Figure 1. Control batter had more air bubbles, and their sizes were larger than egg‐free batters. Addition of SL to cake batter resulted in the formation of more air bubbles having larger sizes. This behavior is due to the emulsifying properties of lecithin resulting in reduction of interfacial tension in liquid and gas phases and formation and stabilization of more air bubbles (Sahi & Alava, 2003; Turabi, Sumnu, & Sahin, 2008).

**Table 1:** Influence of egg substitution on physical properties of batter and cake

Treatment	Batter density (g/cm^3^)	Cake density (g/cm^3^)	Cake height (cm)	Cake volume (cm^3^)
Control	1.028 ± 0.008^d^	0.333 ± 0.009^d^	4.65 ± 0.25^a^	249.37 ± 4.09^a^
SM + 0% SL	1.104 ± 0.013^a^	0.426 ± 0.013^a^	2.80 ± 0.12^e^	194.67 ± 2.89^d^
SM + 1% SL	1.093 ± 0.008^a^	0.403 ± 0.012^b^	2.99 ± 0.13^d^	206.00 ± 2.65^c^
SM + 2% SL	1.073 ± 0.012^b^	0.395 ± 0.015^b^	3.03 ± 0.12^d^	210.33 ± 3.51^c^
SM + 3% SL	1.059 ± 0.010^bc^	0.355 ± 0.012^c^	3.46 ± 0.04^c^	233.33 ± 3.54^b^
SM + 4% SL	1.044 ± 0.011 ^cd^	0.327 ± 0.005^d^	4.46 ± 0.06^b^	247.33 ± 3.05^a^
SM + 5% SL	1.033 ± 0.006^d^	0.323 ± 0.007^d^	4.56 ± 0.05^ab^	251.67 ± 3.78^a^
SM + 6% SL	1.035 ± 0.007^d^	0.324 ± 0.010^d^	4.59 ± 0.06^ab^	250.33 ± 2.06^a^

Mean values ± standard deviation; values followed by different letters in the same column are significantly different (*p* < .05).

### 3.2 Batter viscosity

Determination of rheological properties in cake batter is very important because the quality characteristics of cake such as texture and volume are related to rheological properties of batter (Sakiyan, Sumnu, Sahin, & Bayram, 2004; Turabi et al., 2008). It was found that cake batters exhibited shear thinning behavior and the power law model was found to provide a good fit to the experimental data:

$$\tau =k\cdot \gamma^{n}$$

where τ is the shear stress (pa), γ is the shear rate (per s), *k* is the consistency index (Pa.s^n^), and n is the flow behavior index.

**Figure 2:**
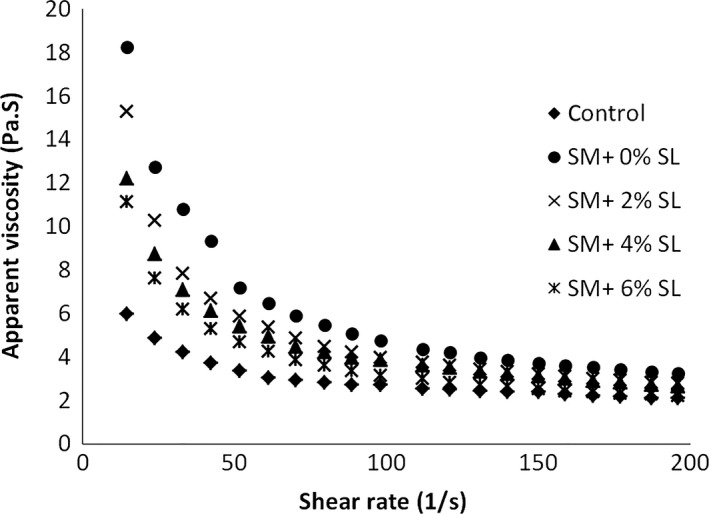
Effects of egg replacement and lecithin level on apparent viscosity of cake batter

A decrease in apparent viscosity with applied shear rate was observed for all cake batters, and the control batter was found to have the lowest apparent viscosity (Figure 2). The equation parameters (k and n) for different batter formulations obtained by fitting the power law model to the experimental data are presented in Table 2. The rheological properties of batters were significantly increased by addition of SM and could be explained by its high water affinity. SM can entrap more water and results in higher viscosity. The addition of SL to batter formulation decreased the apparent viscosity and consistency index. The changes were greater with increasing the SL level due to the increase in air entrapment in cake batters (Sakiyan et al., 2004) and also the reduction in friction between batter particles in the presence of SL. In samples which contain sugar, moisture is adsorbed by sugar particles and when lecithin is added to batter systems the hydrophilic functional groups of lecithin attach to the surface of sugar particles. However, the lipophilic groups are exposed to the surrounding oil phase; hence, the batter particles can slip over each other more easily (Rahmati & Mazaheri Tehrani, 2014). The consistency index also decreased significantly with an increase in the SL level but egg replacement did not affect the flow behavior indices of cake batters as this parameter is generally independent of the formulation (Ilicali, 1985).

**Table 2:** Influence of egg substitution on rheological properties of batter

Treatment	*n*	K (Pa.s^n^)	*R* ^2^
Control	0.450 ± 0.006^a^	32.160 ± 0.707^e^	.979
SM + 0% SL	0.401 ± 0.030^c^	63.655 ± 1.200^a^	.953
SM + 2% SL	0.355 ± 0.063^d^	59.360 ± 0.930^b^	.982
SM + 4% SL	0.419 ± 0.052^b^	51.690 ± 0.649^c^	.995
SM + 6% SL	0.453 ± 0.005^a^	47.620 ± 4.13^d^	.994

Mean values ± standard deviation; values followed by different letters in the same column are significantly different (*p* < .05).

### 3.3 Textural properties of batter

The textural properties of the batters are shown in Table 3. The control cake batter had the lowest textural parameters, while the egg‐free sample without SL had the highest values for all the measured textural parameters (firmness, cohesiveness, consistency, and index of viscosity). Firmness is defined as the maximum force required for the penetration of extrusion cell into the specimen. The higher values indicate that the samples are firmer. Consistency is the positive area under the extrusion graph; the larger the area, the denser the sample. The negative region of the graph is drawn when the extrusion probe returns. The cake batter is a sticky substance; thus, it is lifted with the extrusion probe. This negative area is defined as viscosity index and depends on the weight of sample lifted by the surface of probe. The maximum negative force is taken as a measurement of the cohesiveness of the batter. Generally, sticky samples have higher cohesiveness values in back extrusion test. The significant enhancement of textural parameters in samples with SM is mainly due to the high water absorption of SM that reduces the amount of free water and the mobility of batter particles. It is well documented that ingredients with high water absorption capacity increase the textural parameters of cake batter (Majzoobi, Vosooghi Poor, Jamalian, & Farahnaky, 2016). With increasing concentration of SL, all the textural parameters of batters were decreased significantly (*p* < .05). The addition of SL increased the movement of batter particles and facilitated the penetration and extraction of extrusion probe, thus led to reduction in textural parameters.

**Table 3:** Influence of egg substitution on textural parameters of cake batter and cake

Treatment	Textural parameters of cake crumb				Textural parameters of batter			
	Hardness (g)	Cohesiveness	Springiness	Chewiness (g)	Firmness (g)	Consistency (g.s)	Cohesiveness (g)	index of viscosity (g.s)
Control	242 ± 17.09^e^	441 ± 21.60^f^	412 ± 13.75^f^	294 ± 8.08^f^	359 ± 10.50 ^g^	0.723 ± 0.007^a^	0.915 ± 0.002^a^	237 ± 5.14^ef^
SM + 0% SL	502 ± 28.62^a^	1227 ± 19.47^a^	962 ± 12.50^a^	592 ± 13.11^a^	604 ± 14.42^a^	0.597 ± 0.005^b^	0.845 ± 0.006^e^	304 ± 5.83^a^
SM + 1% SL	483 ± 20.81^a^	1209 ± 10.54^a^	914 ± 15.13^b^	542 ± 22.01^b^	564 ± 12.28^b^	0.589 ± 0.010^b^	0.853 ± 0.004^d^	283 ± 4.17^b^
SM + 2% SL	419 ± 18.00^b^	1078 ± 14.29^b^	890 ± 9.54^b^	473 ± 15.57^c^	522 ± 11.02^c^	0.602 ± 0.009^b^	0.858 ± 0.006^d^	269 ± 3.16^c^
SM + 3% SL	376 ± 22.37^c^	900 ± 11.50^c^	807 ± 19.56^c^	418 ± 14.11^d^	462 ± 17.21^d^	0.600 ± 0.017^b^	0.880 ± 0.003^c^	244 ± 8.27^d^
SM + 4% SL	320 ± 19.52^d^	807 ± 12.64^d^	714 ± 12.77^d^	383 ± 11.59^e^	421 ± 9.07^e^	0.604 ± 0.010^b^	0.900 ± 0.005^b^	228 ± 9.34^e^
SM + 5% SL	316 ± 18.17^d^	768 ± 28.47^e^	661 ± 28.83^e^	360 ± 19.14^e^	392 ± 5.86^f^	0.599 ± 0.010^b^	0.902 ± 0.002^b^	211 ± 3.97 ^g^
SM + 6% SL	317 ± 20.53^d^	761 ± 26.10^e^	639 ± 14.05^e^	377 ± 16.09^e^	397 ± 9.50^f^	0.601 ± 0.009^b^	0.902 ± 0.003^b^	215 ± 4.67 ^g^

Mean values ± standard deviation; values followed by different letters in the same column are significantly different (*p* < .05).

### 3.4 Cake volume and density

During baking, the cake batter is converted to a porous structure basically due to starch gelatinization and protein coagulation depending on starch and protein sources (Roos, 1995). When batter is heated, the air bubbles entrapped in cake batter, expand and lead to an increase in cake volume. The whipping properties of egg are determinant in the cell structure, final volume, and tenderness of cake (Pyler, 1988). The results of cake volume, density, and height are shown in Table 1. An undesirable decrease in volume and height of egg‐free cakes was observed because a too highly viscous batter can restrict its expansion during baking (Sahi & Alava, 2003). Also batters with high density do not have enough air bubbles and restrict cake expansion (Majzoobi, Pashangeh, & Farahnaky, 2014). Inclusion of up to 4% SL to cake formulation increased the cake volume and height. This may be explained by the emulsifying properties of lecithin. During batter mixing, emulsifiers aid the incorporation of air bubbles into the batter and promote foam formation. However, higher levels of SL were not effective in this regard.

### 3.5 Cake texture

The influence of egg substitution on textural properties of cakes can be observed in Table 3. The results showed that when egg was replaced by SM, the hardness and chewiness of cakes were increased while cohesiveness and springiness were decreased. Inclusion of SL to cake formulation improved the textural parameters. Cake texture is affected by density of batter and baked cake, which are related to the amount of air entrapped in batter (Majzoobi, Pashangeh, & Farahnaky, 2013; Sahi & Alava, 2003). Lecithin led to incorporation of more air bubbles in batter and resulted in higher volume and softer texture in cakes. Cohesiveness was not affected by the inclusion of lecithin in cake formulation. This parameter shows the extent of deformation in a sample prior to its rupture. The higher cohesiveness value in control cake was mainly due to the ability of eggs to produce a strong internal network (Rahmati & Mazaheri Tehrani, 2015); however, the samples with SM were more susceptible to mechanical stress and their internal structure was disintegrated by the compression force of the TPA probe. Springiness is an indication of the ability of a sample to recover its original height after the mechanical stress is removed. The springiness in cake crumb is associated with the aggregation of proteins (Wilderjans, Luyts, Goesaert, Brijs, & Delcour, 2010), and the results of this study revealed that incorporation of lecithin increased the springiness of egg‐free samples. Chewiness shows the energy required for chewing a semi‐solid food and is associated with the hardness of the sample; therefore, this parameter was increased in samples with SM and addition of SL decreased this value.

### 3.6 Cake color

The color analysis results are presented in Table 4. Differences were observed between the color parameters of crust and crumb of the control and egg‐free cakes. Determination of the crumb color showed that in control cake, the b‐value was higher but a‐value was lower than egg‐free cake without lecithin. In the presence of lecithin, both a‐values and b‐values were increased, while the L‐value was decreased. This means that the crumb became more reddish, yellowish, and darker. The ingredients used in cake formulation affect the crumb color (Majzoobi et al., 2012). SL has a dark color; thus, inclusion of SL led to a darker crumb color. In terms of crust color, control cake had higher a, b, and L values compared to egg‐free cake without lecithin. Millard reaction, interaction between reducing sugars and amino acids, and caramelization reactions of sugars determine the crust color. Higher color development in egg‐free cakes can be related to the high amount of protein in soy undergoing Maillard reactions and also decrease water mobility due to high water affinity in SM.

**Table 4:** Influence of egg substitution on color parameters of cake crust and crumb

Treatment	Crust color			Crumb color		
	L	a	b	L	a	b
Control	45.67 ± 1.15^a^	23.33 ± 0.58^a^	28.00 ± 3.00^bc^	68.00 ± 2.00^a^	−4.67 ± 0.58^e^	38.00 ± 2.65^b^
SM + 0% SL	32.67 ± 2.08^c^	9.67 ± 1.15^e^	17.67 ± 1.53 ^g^	65.67 ± 2.50^ab^	5.00 ± 1.00^bcd^	19.67 ± 1.15^e^
SM + 1% SL	32.33 ± 1.15^c^	10.67 ± 1.53^e^	18.67 ± 1.50 ^fg^	64.67 ± 1.53^ab^	3.33 ± 2.65^d^	21.67 ± 1.15^e^
SM + 2% SL	32.33 ± 2.08^c^	13.00 ± 1.00^d^	21.67 ± 1.53^ef^	65.00 ± 2.00^ab^	4.33 ± 1.16 ^cd^	25.00 ± 1.00^d^
SM + 3% SL	35.33 ± 1.53^c^	15.33 ± 1.54^c^	24.33 ± 1.53^de^	64.33 ± 2.10^ab^	6.00 ± 1.00^abc^	32.67 ± 2.08^c^
SM + 4% SL	40.33 ± 3.51^b^	16.00 ± 1.00^c^	26.33 ± 1.15 ^cd^	62.33 ± 2.08^b^	6.00 ± 1.73^abc^	40.00 ± 1.00^b^
SM + 5% SL	42.00 ± 1.00^b^	17.00 ± 1.00^bc^	30.33 ± 1.53^ab^	54.00 ± 2.00^c^	7.00 ± 1.00^ab^	45.00 ± 1.73^a^
SM + 6% SL	41.67 ± 0.58^b^	18.67 ± 0.58^b^	32.33 ± 1.53^a^	54.67 ± 1.53^c^	7.67 ± 1.15 ^a^	45.33 ± 2.08^a^

Mean values ± standard deviation; values followed by different letters in the same column are significantly different (*p* < .05).

### 3.7 Sensory attributes

The results of sensory evaluation of different cake samples are shown in Table 5. The sensory and physicochemical properties of foods are mostly dependent on crumb structure due to its effects on appearance, volume, and the textural properties of baked goods (Zghal, Scanlon, & Sapirstein, 2002). The egg‐free cake without SL had firm and dense texture and also inferior appearance and received low scores. The incorporation of up to 4% SL improved all the sensory attributes of egg‐free cakes, and this sample received the same scores as the control cake. However, the unpleasant effects of soy lecithin increased to noticeable levels as SL increased to 5%; these effects are mainly due to the dark color and unpleasant taste of soy lecithin and are reflected in the decrease in the crumb color, taste, and overall quality scores.

**Table 5:** Influence of egg substitution on sensory attributes of cake

Treatment	Taste	Crust color	Crumb color	Texture	Overall acceptability
Control	4.30 ± 0.10^a^	4.64 ± 0.15^a^	4.30 ± 0.10^a^	4.7 ± 0.10^a^	4.50 ± 0.20^a^
SM +0% SL	3.84 ± 0.25^bc^	3.13 ± 0.41^c^	3.90 ± 0.36^b^	2.47 ± 0.25^e^	2.96 ± 0.15^d^
SM + 1% SL	4.20 ± 0.30^ab^	4.0 ± 0.20^b^	4.03 ± 0.25^ab^	3.5 ± 0.36^d^	4.0 ± 0.15^bc^
SM + 2% SL	4.27 ± 0.25^a^	4.50 ± 0.20^a^	4.2 ± 0.10^ab^	4.0 ± 0.12^c^	4.07 ± 0.21^bc^
SM + 3% SL	4.33 ± 0.30^a^	4.67 ± 0.15^a^	4.27 ± 0.15^a^	4.1 ± 0.26^bc^	4.11 ± 0.10^bc^
SM + 4% SL	4.30 ± 0.17^a^	4.56 ± 0.10^a^	4.10 ± 0.10^ab^	4.53 ± 0.15^a^	4.40 ± 0.20^ab^
SM + 5% SL	3.66 ± 0.15^c^	4.46 ± 0.29^a^	3.40 ± 0.10^c^	4.47 ± 0.15^ab^	3.87 ± 0.32^c^
SM + 6% SL	2.77 ± 0.25^d^	4.63 ± 0.25^a^	2.53 ± 0.15^d^	4.36 ± 0.15^abc^	2.8 ± 0.35^d^

Mean values ± standard deviation; values followed by different letters in the same column are significantly different (*p* < .05).

## 4 CONCLUSION

According to the results of this study, egg‐free cake can be prepared using soy products, it may help food manufacturers to produce cakes with low cost and health effects, which are very rich in the amino acid lysine and are useful for the people with specific dietary restrictions or needs such as high cholesterol people and vegetarians. Among different levels of SL tried, 4% was the best as it improved the physicochemical and organoleptic properties of egg‐free cake, and its final quality was close to the control sample. However, when the SL percentage increased the decrease in sensory scores of cake was observed.

## 5 CONFLICT OF INTEREST

None declared.
